# Natural and bioinspired nanostructured bactericidal surfaces

**DOI:** 10.1016/j.cis.2017.07.030

**Published:** 2017-10

**Authors:** Abinash Tripathy, Prosenjit Sen, Bo Su, Wuge H. Briscoe

**Affiliations:** a School of Chemistry, University of Bristol, Cantock's Close, Bristol BS8 1TS, UK; b Centre for Nano Science and Engineering, Indian Institute of Science, Bangalore 560012, India; c School of Oral and Dental Sciences, University of Bristol, Bristol BS1 2LY, UK

## Abstract

Bacterial antibiotic resistance is becoming more widespread due to excessive use of antibiotics in healthcare and agriculture. At the same time the development of new antibiotics has effectively ground to a hold. Chemical modifications of material surfaces have poor long-term performance in preventing bacterial build-up and hence approaches for realising bactericidal action through physical surface topography have become increasingly important in recent years. The complex nature of the bacteria cell wall interactions with nanostructured surfaces represents many challenges while the design of nanostructured bactericidal surfaces is considered. Here we present a brief overview of the bactericidal behaviour of naturally occurring and bio-inspired nanostructured surfaces against different bacteria through the physico-mechanical rupture of the cell wall. Many parameters affect this process including the size, shape, density, rigidity/flexibility and surface chemistry of the surface nanotextures as well as factors such as bacteria specificity (e.g. gram positive and gram negative) and motility. Different fabrication methods for such bactericidal nanostructured surfaces are summarised.

## Highlights

• Several natural nanostructured surfaces can kill bacteria in contact with them.
• Bactericidal efficacy of bio-inspired nanostructured surfaces has been demonstrated.
• Contact killing mechanism of bacteria on nanostructured surfaces not understood
• Optimal geometry and density of nanostructures for killing bacteria unknown
• Cost effective fabrication for large area bactericidal surfaces is important.

## Graphical abstract

**Figure:**
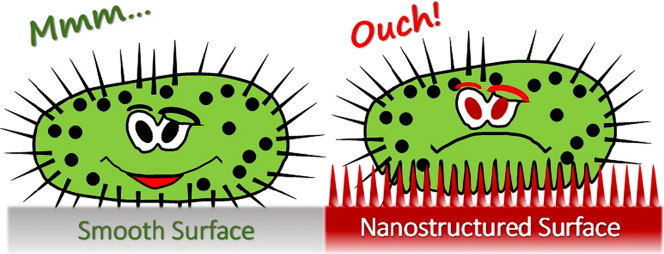

## 1 Introduction

There has been a constant drive for smart technology towards development of materials and surfaces capable of repelling or killing pathogenic microorganisms present on various exteriors in our daily life (such as mobile phones, hospital tools, food packages, kitchen and bathroom surfaces etc.). Most of these surfaces are not intrinsically bactericidal and modifications are thus required for microorganism destruction and prevention of further bacterial infections.

**Fig. 1:**
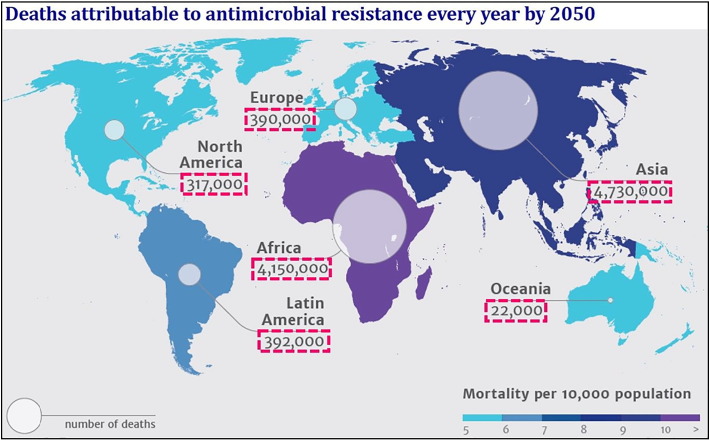
Global distribution of 10 million deaths expected by 2050 due to antimicrobial resistance.

Furthermore, bacterial biofilm formation can be inhibited if the bacteria adhesion and growth can be prevented on the surface in the initial stage [1]. Once a biofilm begins to form, tackling bacterial colonies becomes considerably harder [2]. Whenever an antibiotic is applied to a typical biofilm population, its efficacy in killing the bacteria is limited to the top layer of the biofilm, with little effect on the bacteria located deeper within the microcolonies [3]. Such inabilities of antibiotic agents to penetrate into and exert their effects throughout the biofilm could allow bacterial colonies to develop antibiotic resistance over prolonged periods of use, which is one of the major causes for the failure of using antibiotics against the biofilms [4], [5], [6], [7], [8], [9], [10], [11]. Antimicrobial-resistant infections currently claim 700,000 lives each year from all across the world and this figure will increase alarmingly to 10 million by 2050 if it is not stopped (Fig. 1). One of the methods to tackle biofilms therefore involves prevention of biofilm formation by actively killing the bacteria as soon as they arrive on the surface. Use of antibiotic (chemically) coated surfaces has a significant concern, as widespread antibiotic usage has been linked to the emergence of several multi-drug resistant strains of infectious diseases, some of which (e.g. Tuberculosis) may be epidemic. Many of antibacterial surfaces are effective only in the presence of an aqueous solution, and may prove less effective killing airborne bacteria in the absence of a liquid medium [12].

(Source: The Review on Antimicrobial Resistance – *Tackling drug resistant infections globally*, 2014).

Consequently, instead of killing bacteria chemically, several studies have explored alternative physical methods through the *contact killing mechanism*. These developments have in part been inspired by nature where several insects are known to have bactericidal surfaces that kill microbes coming in contact with them. The bactericidal effects of these surfaces are due to the presence of sharp nanostructures (nano-pillar shaped with diameter 50–250 nm, height 80–250 nm, and pitch 100–250 nm) which pierce into the bacterial cell wall upon contact or rupture the bacteria cell wall, thereby killing the bacteria. Such a physical bactericidal method has become an attractive approach to potentially tackle multi-antibiotic resistant bacteria [13].

Killing bacteria physically though nanostructures rather than chemical means has since become very topical, and several recent reviews on antimicrobial surfaces have focused on different types of antimicrobial coatings to prevent infections [14], [15], use of nanoparticles as antimicrobial agents [16], antimicrobial surfaces based on polymers [17] and other smart materials [18], [19], [20], [21], and naturally occurring antimicrobial surfaces [22], [23]. More generally, nanoparticle dispersions (nanofluids) [24], [25], [26] and nanostructured surfaces are increasingly found in modern formulations and technological applications for controlled adhesion or friction [27], [28], [29] and for enhanced or additional performance and functionalities [30]. Furthermore, the knowledge of nanostructure-bacteria interactions is also intimately related to the topic of nanotoxicity [31] and to our fundamental understanding of interactions between nanoparticles and organised soft matter [32], [33], [34]. However, our knowledge on the design strategies for fabricating effective and economically viable bactericidal nanostructured surfaces remains limited. In this review, we highlight the recent progress on the bactericidal efficacy of different natural and bio-inspired nanostructured surfaces, focusing on the understanding of interactions between nanostructures and the bacteria cell wall, the essential design parameters for efficient nanostructured bactericidal surfaces, and the feasibility of large scale cost-effective fabrication of bactericidal nanostructured surfaces.

## 2 Bacteria cell wall classification

**Fig. 2:**
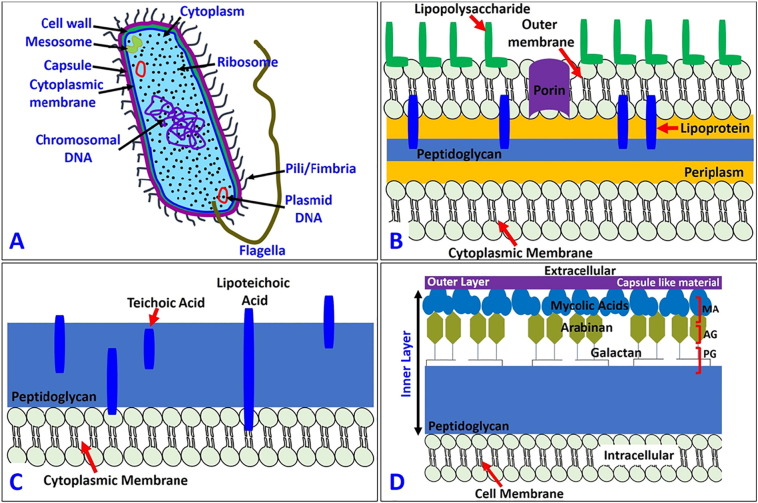
Diagram of the basic components of (A) prokaryotic cell structure, (B) gram negative bacteria, (C) gram positive bacteria and (D) mycobacteria.

The physical killing mechanisms are underpinned by the deformation or rupture of the bacterial cell wall (Fig. 2A), which is a multi-layered structure to provide strength, rigidity, and shape and to protect the microbe from osmotic rupture and mechanical damage [35], [36], [37]. According to their structure, components, and functions, the bacteria cell wall can be divided into the two main categories: gram positive and gram negative. Some bacteria commonly used in antimicrobial research are listed in Table 1, along with their size, source, morphology, and infections they cause.

**Table 1:** Common pathogenic bacteria used in antimicrobial research.

Bacteria	Size	Morphology	Source	Infections
*Gram negative*				
*E. coli*	2 μm long, 0.25–1 μm diameter	Rods	Gastrointestinal tract, animals	Diarrhoea, urinary tract, food poisoning, sepsis
*P. aeruginosa*	1.5–3 μm long, 0.5–0.8 μm diameter	Rods	Water, soil	Infections in immune-compromised hosts, Cystic Fibrosis
*P. fluorescens*	1–3 μm long, 0.5–0.7 μm diameter	Rods	Plants, soil, water surfaces	Affects patients with immune-compromised systems (e.g. cancer patients)
*K. pneumoniae*	2 μm long, 0.5 μm diameter	Rods	Mouth, skin, intestinal tract	Upper and lower respiratory tract infections, renal and urinary tract infections, gastrointestinal tract infections, skin and wound infections, septicaemia
*P. gingivalis*	1.5 μm long, 1 μm diameter	Rods	Gastrointestinal tract, respiratory tract, colon, oral cavity	Pathogenesis of periodontitis
*Salmonella*	2–5 μm long, 0.7–1.5 μm diameter	Rods	Eggs, meat, poultry	Typhoid fever, food poisoning, gastroenteritis, enteric fever
*Campylobacter*	~ 4 μm long, 0.2 μm to 0.5 μm in width	Comma or s-shaped	Raw and undercooked poultry, unpasteurized milk, contaminated water	Diarrhoea, cramps, fever, vomiting

*Gram positive*				
*S. aureus*	0.6 μm cell diameter	Coccal	Nose, respiratory tract, on the skin	Abscesses, sinusitis, food poisoning
*B. subtilis*	4–10 μm long, 0.25–1 μm in diameter	Rod	Soil, gastrointestinal tract	Ear infection, meningitis, urinary tract infection
*E. faecalis*	0.6–2 μm by 0.6–2.5 μm	Coccal	Gastrointestinal tract	Urinary tract infection, endocarditis, septicaemia
*Listeria*	0.5–2 μm long, 0.5 μm in diameter	Rod	Uncooked meats and vegetables. Raw milk, cheese, foods prepared from unpasteurized milk	Listeriosis (food poisoning)
*Clostridium perfringens*	3–8 μm long, 0.4–1.2 μm in diameter	Rod	Intestines of humans and animals, raw meat and poultry	Abdominal pain, stomach cramps, diarrhoea. Nausea

The gram negative cell wall is composed of an outer membrane linked by lipoproteins to thin, mainly single-layered peptidoglycan (PG) (7–8 nm) located within the periplasmic space between the outer and inner membranes. The outer membrane contains the porin, a protein which allows the passage of small hydrophilic molecules across the membrane, and lipopolysaccharide (LPS) molecules that extend into extracellular space. These components in the outer membrane are essential for the structural integrity and viability of gram negative bacteria (Fig. 2B). The cytoplasmic membrane of gram-positive cells contains a thick (30–100 nm) PG layer (4–5 times thicker than that of gram negative bacteria) (Fig. 2C), which is attached to teichoic and lipoteichoic acids that are unique to the gram-positive cell wall. Teichoic acids are attached and embedded in the PG layer, whereas lipoteichoic acids are extended into the cytoplasmic membrane. The gram negative cell wall is more complex, both structurally and chemically [38].

It is also worth mentioning the mycobacteria cell wall, which is thicker than in many other bacteria. Mycobacteria are known to cause many serious diseases such as tuberculosis, leprosy etc. It consists of an inner layer and an outer layer that surround the plasma membrane [39], [40], [41], [42]. The outer layer consists of both proteins and lipids, which are associated with some long- and short-chain fatty acids in the cell wall. The inner layer consists of PG, arabinogalactan (AG), and mycolic acids (MA) covalently linked with each other to form a hydrophobic MA-AG-PG complex (Fig. 2D). The distinguishing characteristic of all mycobacterium is the cell wall which is thicker than in many other bacteria, and it is hydrophobic, waxy, and rich in mycolic acids (Fig. 2D). This kind of cell wall architecture of the mycobacterium protects it in the difficult survival situations. We refer readers to a number of papers in this area for more detailed information [35], [36], [37], [38], [39], [40], [41], [42]. Till now there has not been any study on the bactericidal efficiency of nanostructured surfaces against mycobacterium. This represents an unexplored avenue for future studies in the field of antimicrobial surfaces.

## 3 Naturally occurring nanostructured bactericidal surfaces

Antibacterial surfaces are widespread in nature. There are many plants and insects with antimicrobial surfaces which protect them from pathogenic bacteria. Table 2 lists some of the naturally occurring (as well as artificial mimetic) nanostructured bactericidal surfaces. These bactericidal surfaces typically consist of nanopillars of diameter ~ 50–250 nm, with different heights and densities. A number of early studies have focused on the connection between surface wettability and anti-biofouling effects [43], [44], [45], [46], [47], [48], [49], [50], [51], [52], [53], attributing it to non-stickiness of the microbes on the presumed superhydrophobic surface. That is, hydrophilic surfaces seem to allow bacteria proliferation, whereas hydrophobic surfaces prohibit bacterial growth as the bacteria cannot stick to the surface. Surface hydrophobicity or superhydrophobicity is more critical in water-immersed conditions (entailing air entrapment) than in air. More recent observations show that such natural nanostructured surfaces can kill bacteria by rupturing the cell wall, known as the contact killing mechanism [54].

**Table 2:** Summary of naturally occurring and artificial nanostructured bactericidal surfaces.

Surface	SEM image	Surface features	Preparation method	Wettability	Bactericidal efficacy
*Naturally occurring*					
Cicada wing [54]		Nanoneedles, height 200 nm, diameter 60 nm size at the top, 100 nm at the base of the pillar, and spacing 170 nm	Natural	Hydrophobic, water contact angle (CA) = 159°	Lethal to *P. aeruginosa*, gram negative (g − ve)
Gecko skin [56]		Hair (spinules) like structures with sub-micron spacing and a tip radius of curvature < 20 nm	Natural	Hydrophobic, CA = 151°–155°	Lethal to *Porphyromonas gingivalis* (g − ve)
Dragon fly wing [57]		Nanograss, diameter 50–70 nm, height 240 nm	Natural	Hydrophobic, CA = 153°	Lethal to *P. aeruginosa* (g − ve), *S. aureus* (g + ve) and *B. subtilis* (gram positive (g + ve))
Periodical cicada [88]		Hemispherical nano features with height 83.5 nm, diameter 167 nm, pitch 252 nm	Natural	Hydrophilic, CA = 80.1°	Caused cell wall rupturing of *S. cerevisiae* (Yeast)
Annual DD cicada [88]		Spherical nanocones with height 183 nm, base diameter 104 nm, cap diameter 104 nm, pitch 175 nm	Natural	Hydrophobic, CA = 132°	Caused cell wall rupturing of *S. cerevisiae*
Sanddragon dragonfly [88]		High-aspect ratio spherical capped nanocylinders with height 241 nm, diameter 53 nm, pitch 123 nm	Natural	Hydrophobic, CA = 119°	Caused cell wall rupturing of *S. cerevisiae*
Megapomponia intermedia [89]		Nanopillars with height 241 nm, diameter 156 nm, pitch 165 nm	Natural	Hydrophobic, CA = 135.5°	Bactericidal against *P. fluorescens* (g − ve)
Cryptotympana aguila [89]		Nanopillars with height 182 nm, diameter 159 nm, pitch 187 nm	Natural	Hydrophobic, CA = 113.2°	Bactericidal against g − ve *P. fluorescens*
Ayuthia spectabile [89]		Nanopillars with height 182 nm, diameter 207 nm, pitch 251 nm	Natural	Hydrophobic, CA = 95.65°	Bactericidal against g − ve *P. fluorescens* (but more than Megapomponia intermedia and Cryptotympana aguila)

*Bio-inspired and artificial*					
Black silicon [57]		Nanograss, diameter 20–80 nm, height 500 nm	RIE	Hydrophilic, CA = 80°	Lethal to *P. aeruginosa* (g − ve), *S. aureus* (g + ve) and *B. subtilis* (g + ve)
Black silicon [59]		Nanograss, diameter 220 nm, height 4 μm	DRIE	Hydrophobic, CA = 154°	Lethal to *Escherichia coli, Staphylococcus aureus* and mammalian cells (mouse osteoblasts)
Diamond nanocone surface [61]		Nanocones with sharp tips, diameter of tips 10–40 nm, width of nanocones 350 nm- 1.2 μm, height 3–5 μm	RIE	–	Lethal to *P. aeruginosa* (g − ve)
Diamond coated black silicon [62]		High aspect ratio nanoneedles, height 0.5–1 μm (short needle) and 15–20 μm (long needle)	RIE	–	Lethal to *P. aeruginosa* (g − ve)
Titania nanowire arrays [64]		Nanowires Brush type: diameter 100 nm	Hydrothermal process	–	Effective in killing motile bacteria (*P. aeruginosa*, *E. coli* and *B. subtilis*), Less lethal against non-motile bacteria (*S. aureus*, *E. faecalis*, *and K. pneumoniae*)
Titania nanowire arrays [64]		Nanowires Niche type: diameter 10–15 μm	Hydrothermal process		Effective in killing motile bacteria (*P. aeruginosa*, *E. coli* and *B. subtilis*), Less lethal against non-motile bacteria (*S. aureus*, *E. faecalis*, *and K. pneumoniae*)
Titanium nanopatterned arrays [65]		Nanopatterned arrays, average diameter 40.3 nm	Hydrothermal etching	Hydrophilic, CA = 73°	Effective in killing *P. aeruginosa* (g − ve), Less lethal against *S. aureus* (g + ve)
Ti alloy nanospike surface [66]		Nanospikes, average diameter 10 nm, spacing 2 μm, height 2 μm	Anodization	–	Lethal to *S. aureus*
Ti alloy nanospike surface [67]		Nanospikes, average diameter 20 nm	Thermal oxidation	–	Lethal to *E. coli*
Nanopatterned polymer surface [68]		PMMA nano pillar surfaces, diameter 70–215 nm, and height 200–300 nm	Nanoimprint lithography	–	Showed lethal action against *E. coli*
Nanostructured PMMA film [69]		Nanopores, depth 460 nm, spacing 300 nm, aspect ratio 3.0	Nanoimprint lithography	Hydrophobic, CA = 114.5°	Restricted attachment of bacterial and mammalian cells
Structured polystyrene surface [70]		Line structure, width of line 1.63 μm, period 5 μm (note: these are micro- not nano-structures)	Direct laser interference patterning	–	Enhanced *S. aureus* (g + ve) adhesion
Structured polystyrene surface [70]		Pillar structure, diameter of pillar 1.85 μm, period 5 μm (note: these are micro- not nano-structures)	Direct laser interference patterning	–	Enhanced *S. aureus* (g + ve) adhesion
Structured polystyrene surface [70]		Lamella structure, width of lamella 0.47 μm, period 2 μm (note: these are micro- not nano-structures)	Direct laser interference patterning	–	Showed reduction in *S. aureus* (g + ve) adhesion
Nanostructures sutures [90]		Lamella structure with 100 nm thickness and 500 nm length (after 1 min plasma treatment)	Plasma etching	–	Effective in preventing adhesion of *E. coli* (g − ve) and biofilm formation
Au nanostructured surface [91]		Au nanopillars (diameter 50 nm, height 100 nm)	Electrodeposition and plasma etching	–	Lethal to *S. aureus* (g + ve)
Au nanostructured surface [91]		Au nanorings (diameter 100–200 nm, height 100 nm)	Electrodeposition and plasma etching	–	Lethal to *S. aureus* (g + ve)
Au nanostructured surface [91]		Au nanonuggets (diameter 100–200 nm, height 100 nm)	Electrodeposition and plasma etching	–	Lethal to *S. aureus* (g + ve)

**Fig. 3:**
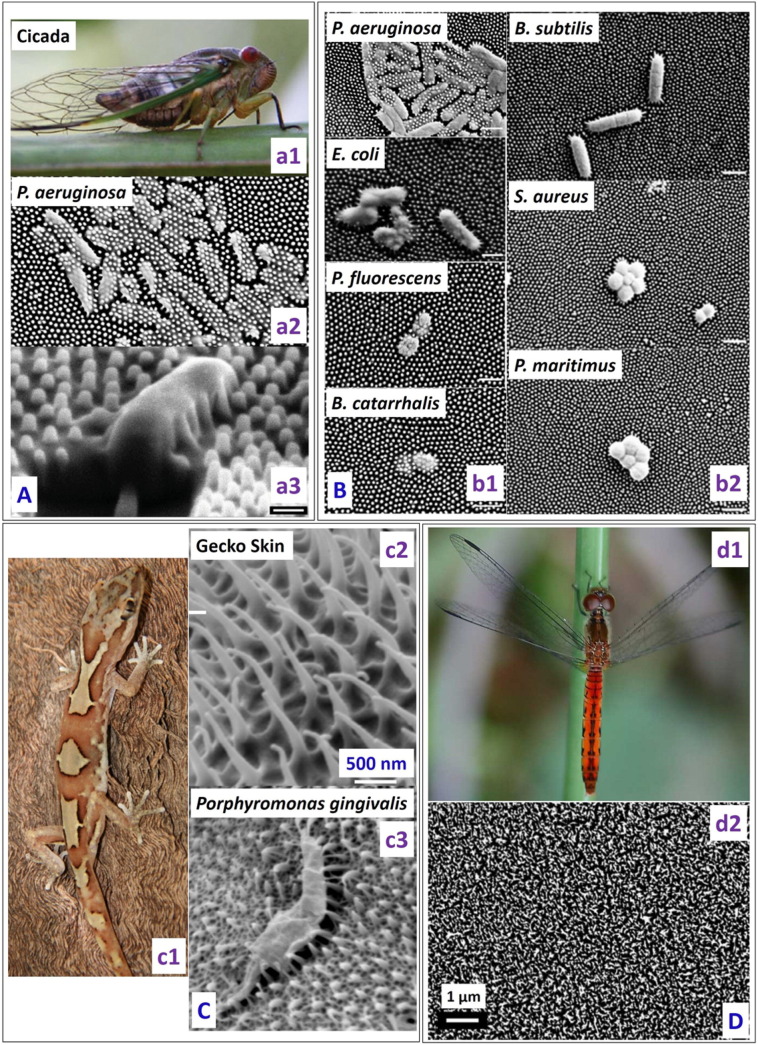
(A) (a1) Photograph of cicada insect (*Psaltoda claripennis*). (a2) *Pseudomonas aeruginosa* cells on the nanostructured cicada wing penetrated by the nanopillar structures. (a3) Representative SEM image of a *Pseudomonas aeruginosa* cell sinking between the nanopillars on the cicada wing surface (53° view angle) (scale bar = 1 μm) [reproduced with permission from Ref. [54]]. (B) Selective bactericidal activity of the Cicada wing surface against (b1) gram negative (*P. aeruginosa*, *E. coli*, *P. fluorescens*, *B. catarrhalis*) and (b2) gram positive bacteria (*B. subtilis*, *S. aureus*, *P. maritimus*) (Scale bars = 1 μm). SEM images depict that there is little effect of the nanostructured cicada wing surface on the size and morphology of gram positive bacteria [reproduced with permission from Ref. [55]]. (C) (c1) Image of the gecko *Lucasium steindachneri* (c2) SEM image of the gecko skin consisting of hair like structures with sub-micron spacing and a radius of curvature < 20 nm. (c3) SEM image of the *Porphyromonas gingivalis* interacting with the nano-structured Gecko skin [reproduced with permission from Ref. [56]]. (D) (d1) Image of a dragonfly (Source: Bill Higham@flickr) and (d2) SEM image showing the random distribution of the nanostructures present on the dragonfly wing [reproduced with permission from Ref. [57]].

One of the first studies of the naturally occurring bactericidal surface of cicada wings against *P. aeruginosa* (gram-negative) was reported in 2012 by Ivanova et al. [54]. The nanocones present on the cicada wing are uniform in height (200 nm), shape (60 nm diameter size at the top and 100 nm at the base of the pillar) and spatial distribution (170 nm apart) (Fig. 3A). In contrast to previous reports, they showed that, despite the superhydrophobic nature of the cicada wing (static water contact angle (CA) of 158.8°), there was significant bacterial adhesion on the nanostructured surface. On contact, the adhered bacteria went through a rapid morphological change and were killed within 5 min as estimated through imaging techniques. It was concluded that the anti-biofouling nature of cicada wings was not due to its ability to repel the bacteria, rather to its ability of kill them upon contact. The kill rate of *P*. *aeruginosa* on cicada wings was approximately 2.05 × 10^5^ colony forming units (cfu) min^− 1^ cm^− 2^. It was also reported that an attachment/kill cycle of 20 min seemed present, during which the wing surface was first saturated with the bacteria which were then killed and dispersed before the next group of bacteria could attach to the surface. Using atomic force microscopy (AFM), the time required for wall rupture was estimated to be approximately 3 min. The wing was also made hydrophilic with a 10 nm gold coating while its surface topography was retained, and it was found that its bactericidal activity was preserved, confirming the physico-mechanical nature of the killing.

However, the cicada wing could only effectively kill gram negative bacteria but not gram positive bacteria [55], which has been attributed to their peptidoglycan cell wall being 4–5 times thicker than that of gram negative bacteria (Fig. 3B). This selective killing of gram negative bacteria is consistent with the mechanical model predicting cell-wall rigidity as the primary factor determining the ability of bacteria to survive the bactericidal cicada wing.

Watson et al. [56] demonstrated the bactericidal nature of a gecko skin with micro-/nano-structures consisting of spinules with a radius of curvature smaller than 20 nm and spacing in the sub-micron range. They found that the gecko skin was lethal to *Porphyromonas gingivalis*, a gram negative, nonmotile, pathogenic bacterium. It was suggested that the bacteria cell wall was stretched and ruptured when it came in contact with the nanostructured Gecko skin (Fig. 3C). However, bactericidal efficacy of gecko skin was not tested against any gram positive bacterium.

Ivanova et al. studied the bactericidal efficacy of the dragonfly wing surface [57]. Unlike cicada wing, the nanostructures present on the dragonfly wing are randomly distributed in terms of shape, size and distribution (Fig. 3D). The nanopillar diameters on dragonfly wings show a sigmoidal distribution below 90 nm. The dragonfly wing was shown to be very efficient in killing both gram negative (*P. aeruginosa*) and gram positive bacteria (*S. aureus* and *B. subtilis*), as well as endospores (*B. subtilis*) with a kill rate of approximately 4.5 × 10^5^ cfu/(minute^∗^ cm^2^).

Hayes et al. [58] described the surface texture of the cuticle of the aquatic larvae of the drone fly. An array of nanopillars (diameter < 100 nm, length 200–1000 nm, average spacing 230 nm) were observed on the cuticle. The surface of the drone fly was found to be hydrophilic unlike the superhydrophobic cicada wing, with the nanopillar density on the cuticle as high as that on a cicada wing [54]. It was suggested that this surface might antagonize the formation of biofilms and would potentially act as an efficient bactericidal surface. However, they did not test the bacteriacidal efficacy of the surface against any pathogenic bacteria.

Ma et al. [49] reported the fouling resistance behaviour of the Taro leaf in both nonwet (fresh leaf without any surface treatment) and wet (leaf underwent soaking treatment/water vapour condensation/ethanol wetting to make it hydrophilic) conditions. *P. aeruginosa* was used for testing the adhesion with the Taro leaf which exhibits superhydrophobicity with a high static water contact angle and low roll off angle due to the presence of the hydrophobic epicuticle layer and the micro/nano structures. Under the nonwet condition, the anti-adhesion property of the Taro leaf towards *P. aeruginosa* cell suspension (concentration ~ 2 × 10^7^ cfu/ml) in PBS was due to the trapped air between the nanostructures. However, the anti-adhesion property observed under the completely wet condition was attributed to the reduced adhesion force in the area of the Taro leaf covered with dense nanostructures, although the exact mechanism for the adhesion reduction was not explained. The surface of the nanostructured Taro leaf was found to be bacteriostatic rather than bactericidal. This finding is useful when considering the design for antimicrobial structures for underwater applications.

## 4 Silicon based nanostructured bactericidal surfaces

**Fig. 4:**
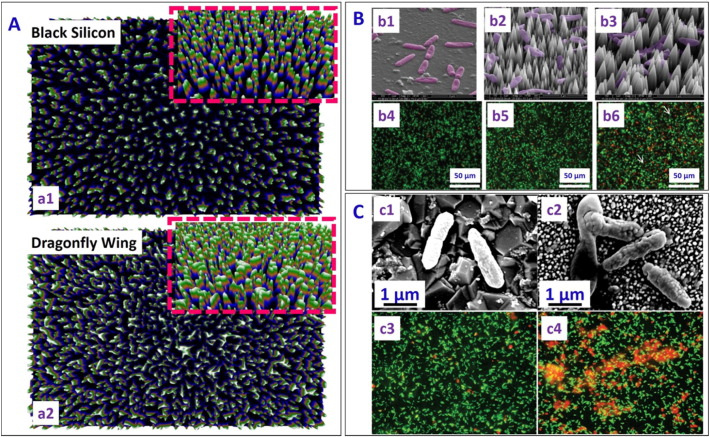
(A) Image highlighting differences and similarities of (a1) black silicon (bSi) and (a2) dragonfly wings created by a three-dimensional reconstructions based on a displacement map technique. Inset shows tilted view at an angle of 53° [reproduced with permission from Ref. [57]]. (B) Representative SEM images of *P. aeruginosa* on (b1) flat silicon control, (b2) high and (b3) low nanocone density diamond coated silicon surface. Fluorescence micrographs of *P. aeruginosa* after 1 h incubation on these surfaces are shown in (b4)–(b6) respectively. More dead cells (appearing red) were observed on the low density nanostructured silicon surface as compared to high density nanostructured silicon surface and flat silicon surface [reproduced with permission from Ref. [61]]. (C) SEM images of (c1) healthy *P. aeruginosa* on flat boron-doped diamond control surface, and (c2) damaged bacteria cells on black silicon sample coated with diamond after 1 h of incubation. Fluorescence micrographs of *Pseudomonas aeruginosa* on (c3) control flat boron-doped diamond surface, and (c4) black silicon sample coated with diamond after 1 h incubation, showing more dead on the nanostructured black silicon as compared to the flat boron doped diamond surface (Red and green colours due to Propidium Iodide and Syto-9 dyes respectively) [reproduced with permission from Ref. [62]].

Surfaces bearing well-defined nanotextures have been increasingly found in modern applications to facilitate enhanced functionalities and desired properties [24], [27], [29]. Due to the ease of fabrication and distinct electronic, optical, mechanical and thermal properties, silicon is widely used in industrial applications. It is so far the surface of choice for mimicking natural bactericidal surfaces, with the focus on reproducing the geometric features and surface chemistry observed on the cicada and dragonfly wings, and also the lotus and taro leaves. To mimic dragonfly wing, Ivanova et al. [57] developed black silicon surfaces using reactive ion etching of silicon. Nanopillar diameters on the black silicon surface showed a bimodal distribution spanning 20–80 nm, with bactericidal efficacy matching that of the dragonfly wing (Fig. 4A). Although such nanotextured black silicon could effectively kill minimum infective doses of *S. aureus* and *P. aeruginosa* in very short time in a nutrient deficient environment, in nutrient rich environment the bacteria could survive up to 6 h. The black silicon was also found to be more efficient in killing both gram positive and gram negative bacteria as compared to the dragonfly wing (Refer Table 2 of Reference 42 for more details).

Hasan et al. [59] fabricated nanostructured silicon surfaces using the deep reactive ion etching (DRIE) technique. The surface with pillars 4 μm tall and 220 nm in diameter were superhydrophobic (static contact angle (CA) 154° and contact angle hysteresis 8.3°), contrasting with the hydrophilic nature of the native black silicon [57]. Bacterial viability studies showed that 83% of gram negative (*E. coli*) and 86% of gram positive (*S. aureus*) bacteria exposed to the surface were killed in 3 h. A fast initial kill rate was noted, with 25% of the bacteria killed in the first 5 min. For this surface, a different killing mechanism has been proposed, based on pinning of the cellular membrane on the nanopillars as the motile cell tries to find other attachment points by stretching themselves. As the bacterial cell reaches their limit of stretching, it ruptures and dies. This different mechanism could be due to the larger diameter and height of the nanopillars in this study in comparison to the previous studies [57].

Exploiting the mechanical hardness, high bulk modulus, and low compressibility of diamond [60], Fisher et al. [61] reported fabrication of nanocone-shaped diamond on silicon substrate with two nanocone densities and tested their bactericidal efficacy. The nanocones were 3–5 μm in height, with sharp tips of diameter 10–40 nm and a base width 350 nm–1.2 μm, achieved by controlling the bias voltage in RIE. These nanoconed diamond surfaces caused significant killing of gram negative *P. aeruginosa* as compared to the control silicon surface (Fig. 4B). The killing efficiency of the surface with a lower nanocone density was found to be 17% higher than that with a higher nanocone density (more dead cell (red colour due to Propidium Iodide Dye and green colour because of Syto-9) seen in Fig. 4B (b6) as compared to Fig. 4B (b5)). This was attributed to the larger spacing between the nanocones which facilitated more extended stretching of the cell membrane, leading to bacteria lysis. May et al. [62] further demonstrated that black silicon surfaces coated with diamond nanoneedles of two different heights (0.5–1 μm and 15–20 μm, respectively) showed excellent bactericidal activity against pathogenic *P. aeruginosa*. Both scanning electron microscopy and fluorescence microscopy images demonstrated the higher bactericidal efficacy of the diamond nanoneedle surface as compared to the flat boron doped diamond control surface (Fig. 4C).

It is however important to note that the bactericidal efficacy of nanostructured surfaces depends on parameters such as the nanostructure dimension, coatings present, and the type and size of bacteria. Indeed, proliferation of cells on different nanostructures under certain conditions has also been reported. For instance, Hizal et al. [63] reported the detachment of bacteria (*Staphylococcus epidermidis* (non-extracellular polymeric substance (n-EPS) producing strain) and *Staphylococcus aureus* (EPS producing strain) from a nanostructured silicon surface (with blunt nanopillars of pitch values 200, 400 and 800 nm) to a smooth surface, due to smaller bacterial adhesion on the nanostructured surface as compared to the smooth surface. This was attributed to the decreased surface area between the textured substrate and the adhering bacterium. No significant bactericidal activity was observed on the nanostructures surfaces, possibly due to the nanopillars not deforming and stretching the cell wall of the spherical bacteria, as observed for rod shaped bacteria. Bacteria were also observed to settle between the pillars in the case of the large pitch value (800 nm).

## 5 Titania based nanostructured bactericidal surfaces

**Fig. 5:**
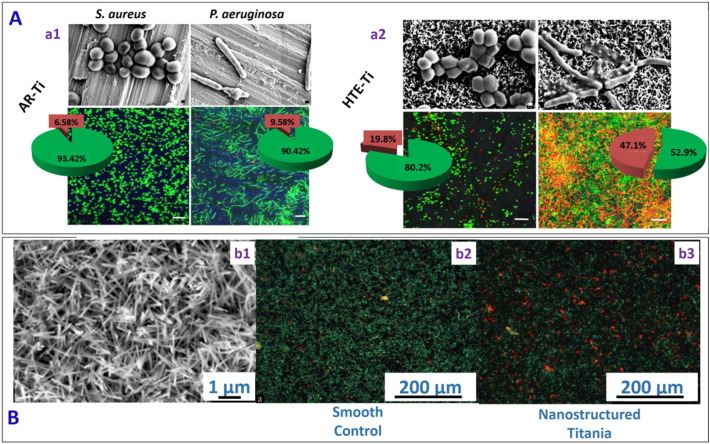
(A) Representative SEM and confocal laser scanning microscopy images of *S. aureus* and *P. aeruginosa* on as received flat titania (AR-Ti) surface (a1), and on hydrothermally etched titania (HTE-Ti) surface mimicking dragonfly wings (a2), the latter showing a higher killing rate (red portions in the pie charts) for both bacteria [reproduced with permission from Ref. [65]]. (B) (b1) SEM images of nanospikes on titania substrate. (b2) Fluorescence microscopy images of *E. coli* on smooth control surface and (b3) nanostructured titania surface. More dead cells were seen on the nanostructured titania surface as compared to the smooth control surface (Red-dead cells, green-live cells) [reproduced with permission from Ref. [67]].

Titania is important in many applications because of its biocompatibility, mechanical stability and chemical inertness. Diu et al. [64] fabricated titania nanostructured surfaces (nanowires with diameter ~ 100 nm) of two different morphologies (brush and niche type) using an alkaline hydrothermal process. They found that the bactericidal effect of the nanostructured surfaces against motile (*P. aeruginosa*, *E. coli* and *B. subtilis*) bacteria was more pronounced than that against non-motile bacteria (*S. aureus*, *E. faecalis* and *K. pneumonia*). Chris et al. [65] fabricated hierarchically ordered Titanium nano-patterned arrays (average diameter ~ 40.3 ± 20.0 nm) mimicking the dragonfly wing using a chemical hydrothermal process at high temperature. The fabricated surfaces allowed the adherence of human cells, but showed excellent bactericidal behaviour against *P. aeruginosa* and *S. aureus* (Fig. 5A). Similarly, Ferdi et al. [66] reported the fabrication of 2D nanoporous (pore diameter 55 nm, depth 1 μm and interpore distance 70 nm) and hierarchical 3D nanopillared surface (average tip diameter 10 nm, height 2 μm, and average distance between nanopillars 2 μm) on titanium, and then a “smart” bacteria-triggered self-defensive coating containing tannic acid/gentamicin was deposited atop via the layer by layer (LbL) technique (see Table 2). The tannic acid/gentamicin coating on the 3D nanopillared surface allowed a greater exposure of the antibiotic coating to the adhering bacteria and increased the antibacterial efficiency by 10-fold compared to a smooth surface coated with the same antibacterial coating, thus allowing a reduction in the number of cycles used in LBL coating deposition for the same killing efficiency. Terje et al. [67] fabricated nanostructured surface (nanospikes with a diameter ~ 20 nm with an anisotropic surface distribution (Fig. 5B (b1)) on Titanium alloy using a thermal oxidation method. They noticed a 40% reduction in *E. coli* viability on the nanospike surface as compared to the smooth control surface (Fig. 5B (b2 & b3)).

## 6 Flexible nanostructured bactericidal surfaces

**Fig. 6:**
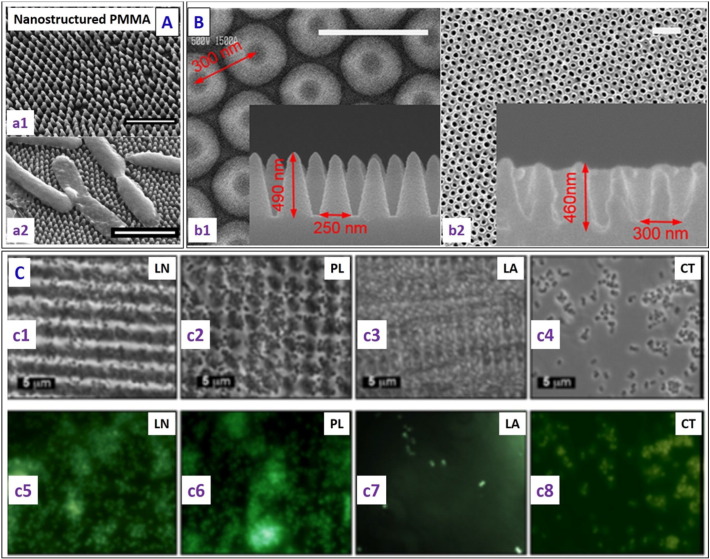
(A) (a1) Representative SEM image of PMMA surfaces at a 30° tilt, where a two-step lithography process was carried out to replicate the nanostructures on the surface of the cicada wing in PMMA (scale bar 1 μm). (a2) Representative SEM micrograph of *E. coli* on patterned PMMA surfaces (scale bar - 2 μm), showing deflated bacteria draped across several PMMA pillars (whereas they retained the rod-shape on the control flat PMMA surface) [reproduced with permission from Ref. [68]]. (B) SEM images of the nanostructured surface: (b1) top view and cross-sectional view (inset) of the silicon master surface, and (b2) nanostructured pattern on the PMMA film and a magnified cross-sectional image (inset) (scale bar – 500 nm) [reproduced with permission from Ref. [69]]. (C) (c1) Attachment of *S. aureus* to patterned PS wafers (c1) LN- line structure, (c2) PL- pillar structure, (c3) LA- lamella structure and (c4) CT- control surface. Corresponding fluorescence microscopic images (bottom) showing more *S. aureus* (green spots showing the number of bacteria present) adhesion on the (c5) line (LN), (c6) pillar (PL) and (c8) control surfaces than the (c7) lamella (LA) structures [reproduced with permission from Ref. [70]].

Nanostructured surfaces with bactericidal behaviour can also be realized on flexible substrates. To mimic cicada wings, Dickson et al. [68] showed that most of the gram negative *E. coli* bacteria incubated on poly (methyl methacrylate) (PMMA) films with nanopillars (diameter 70–215 nm and height 200–300 nm) fabricated using nanoimprint lithography, were killed (Fig. 6A). This reduced the bacterial load in contaminated aqueous suspensions by 50% over a 24 h period as compared to flat controls. The optimal spacing between the nanopillars was reported to be within 130 nm to 380 nm for good bactericidal response.

Kim et al. [69] showed PMMA surfaces with periodic nanostructures (height 460 nm, aspect ratio 3, and spacing ~ 300 nm) fabricated using nanoimprint lithography exhibited hydrophobicity, anti-reflectivity (with < 0.5% reflectance) and antimicrobial properties (Fig. 6B). Valle et al. [70] utilised the technique of direct laser interference patterning (DLIP) to fabricate line- and pillar-like patterns and complex lamella microstructures on polystyrene. These surfaces were tested against gram positive *S. aureus* in both static and continuous culture flow conditions, and it was concluded that the line- and pillar-like patterns enhanced *S. aureus* adhesion, whereas the complex lamella microtopography reduced *S. aureus* adhesion under both flow conditions (Fig. 6C).

## 7 Selectivity and specificity of bactericidal surfaces: interactions of nanostructured surfaces with mammalian, RBC and other cells

**Fig. 7:**
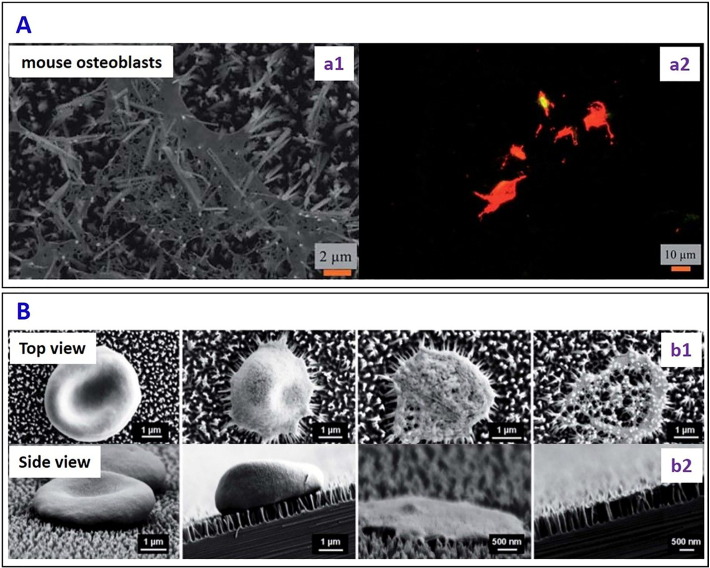
(A) (a1) SEM micrograph and (a2) fluorescent images of a mammalian cell on nanopillars on the silicon surface. Red colour confirmed the lysis of the mammalian cells on the nanopillars [reproduced with permission from Ref. [59]]. (B) The time dependent morphological variation of erythrocytes interacting with the nanopillared black silicon surfaces. SEM images (b1) Top and (b2) Side views showing in stages the morphological changes taking place as an RBC ruptures due to the interaction with the nanopillars [reproduced with permission from Ref. [80]].

A number of recent studies [71], [72], [73], [74], [75], [76], [77], [78], [79] have investigated the parameters affecting the interactions of nanostructured surfaces with other cell walls (cells other than bacteria, e.g. mammalian, red blood cells), and such knowledge is relevant to our considerations of the selectivity of bactericidal surfaces. The nanostructured surface fabricated by DRIE [59] displayed lethal action against mammalian cells (mouse osteoblasts) by mechanically rupturing the cell wall, leading to a 12% viability (Fig. 7A). Pham et al. [80] reported the interaction of the erythrocytes (RBC) with the nanopillars on the black silicon with a tip diameter of ~ 12 nm and a pillar length of ~ 600 nm. The nanopillars caused stress-induced cell deformation, rupture and lysis (Fig. 7B). A model for the interaction of the nanopillars and RBC cell wall was put forward in terms of a free energy driving force, showing that the lysis of the erythrocyte took place because of the piercing of the membrane by the nanopillars present on the black silicon surface.

**Fig. 8:**
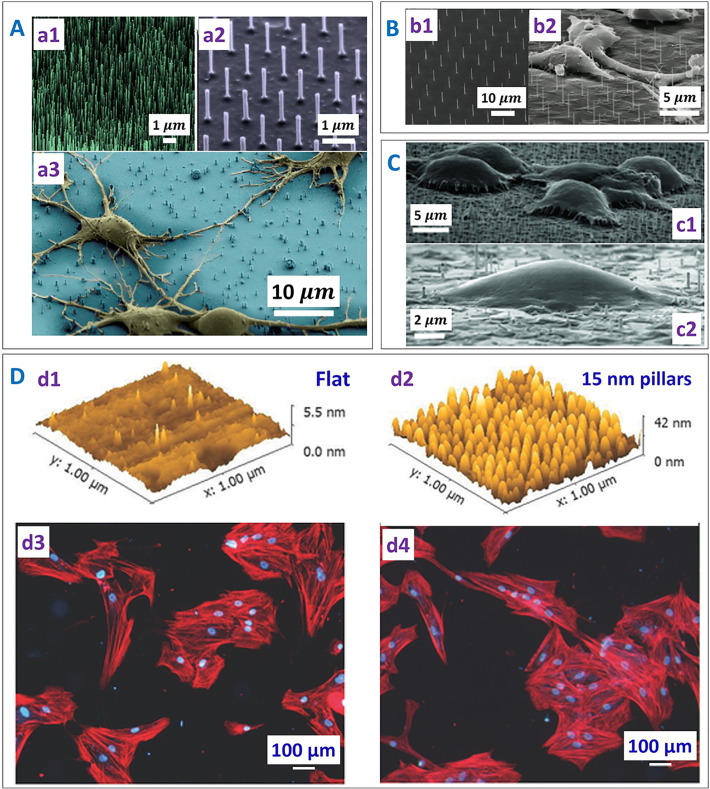
Interactions between cells with nanostructured surfaces. (A) Representative SEM images of silicon nanowires (NWs) fabricated by the (a1) chemical vapour deposition (CVD) and (a2) the reactive ion etching (RIE) method. (a3) SEM image showing the morphology of the deformed rat hippocampal neurons on the silicon nanowires [reproduced with permission from Ref. [71]]. (B) (b1) SEM images of Si nanowires (NWs) fabricated by e-beam lithography and (b2) deformed human embryonic kidney 293 cells due to NW penetration [reproduced with permission from Ref. [73]]. (C) (c1) Fakir state with the cell hanging on top of the nanostructures and (c2) Wenzel state with complete deformation of the cell on the nanostructured array [reproduced with permission from Ref. [77]]. (D) AFM images of (d1) flat and (d2) nanopatterned titania surface. Immunofluorescent images of intact human bone marrow cells on (d3) flat titania control surface and (d4) the surface bearing 15 nm high nanopillars. Human bone marrow cells appeared polarized and elongated on the nanopillared surface as compared to the flat control surface. Also a higher number of cells were counted on the nanopillared surface as compared to the flat titania surface [reproduced with permission from Ref. [78]].

Shalek et al. [71] showed that > 95% of vertical silicon nanowires (NWs) prepared by chemical vapour deposition (CVD) and reactive ion etching (RIE) could penetrate into HeLa cells (Magenta) bedded atop after 1 h incubation, delivering the biomolecules attached to the NWs (Fig. 8A), although the forces involved in the process were not discussed. However, growth and division of the HeLa cells was observed on the silicon NWs despite of the penetration. Berthing et al. [73] used a fluorescence labelling and imaging technique to study the conformation of human embryonic kidney cells on the NW array with single NW resolution (Fig. 8B). It was shown that the outer cell membrane was not penetrated by the NW and instead adapted its conformation to enclose the individual NWs.

Recently in a cell interface with nanostructured arrays (CINA) model, Bonde et al. [77] assumed that the nanotextures (random array: density 7–200 nanostructures/100 μm^2^, length 1–5 μm, diameter 70–200 nm; ordered nanostructures array: spacing 3–5 μm, length 11 ± 2 μm, 5 ± 1 μm, diameter 100 ± 20 nm) deformed the cell wall membrane, rather than penetrating it, with the cells settling on the nanostructured surface in two states: the Fakir state with the cells hanging on top of the nanostructures, and the Wenzel state with the cells completely deformed around the nanostructures and coming in contact with the flat substrate between them (Fig. 8C). The aspect ratio of the nanostructures determines whether the bacteria will be in the Fakir state or the Wenzel state. In the case of high aspect ratio structures, bacteria mostly remain in the Fakir state as they cannot touch the bottom surface. However, in the case of the low aspect ratio, there can be a transition from the Fakir to the Wenzel state as the bacteria can touch the flat bottom surface and settle there. Forces such as gravity and adhesion acting on the cell membrane were discussed. It was suggested that the cell settling mechanism was highly dependent on both the single nanostructure dimension and the nanostructure density. Silverwood et al. [78] found improved bone deposition from a co-culture of human bone marrow cells without unwanted osteoclastogenesis on titanium nanopillars (15 nm in diameter and average spacing ~ 30 nm), as compared with that on flat titanium (Fig. 8D), suggesting they could be used for orthopaedic implant applications. Tsimbouri et al. [79] fabricated titania nanospikes (average diameter 25.1 nm, height 1 μm, randomly oriented) on titanium surface using the hydrothermal process. Such surfaces supported the osteoblast (stem cell) growth and at the same time showed bactericidal behaviour against gram negative *P. aeruginosa*.

Choi et al. [81] studied the interaction between human foreskin fibroblasts cells and two different nanopatterns (nanoposts and nanogratings) fabricated using interference lithography and deep reactive ion etching technique (DRIE) (height 50–600 nm, pitch 230 nm for both nanoposts and nanogratings). Cell proliferation was similar on the 2D smooth surface and short (50–100 nm) 3D nanoposts and nanograting structures. In contrast, cell proliferation was suppressed the on the needle-like nanoposts and nanograting structures with higher textures (200–300 nm and 500–600 nm). While the cells retained their shape on the 2D smooth surface, they exhibited different morphologies on the 3D nanoposts. On the short (50–100 nm high) nanoposts the cells became elongated; on medium height (200–300 nm) nanoposts, the cells elongated and also shrank in size; on tall (500–600 nm high) nanoposts, the cells exhibited a rounded shape with a much smaller size. In contrast, cells were found to spread well on the nanograting structures with more pronounced elongation on the taller nanograting structures as compared to the shorter ones. The results thus again point to the importance of the nanotexture topographic characteristics in determining the cell-substrate interactions.

Ideally, the co-culture model should be used so that the interactions of nanostructured surfaces with bacteria and mammalian cells are studied simultaneously to assess ‘*the race for the surface*’ effect. However, such a model is complex, cell type-dependent and still under development [82], [83], [84], [85]. There are currently few reports on the studies of the nanostructured surfaces using such a co-culture model, and most often the cellular and bacterial interactions with biomimetic nanostructured surfaces are characterized either sequentially [86] or separately [79].

## 8 Effects of physical characteristics of nanostructured surfaces on their bactericidal efficacy

**Fig. 9:**
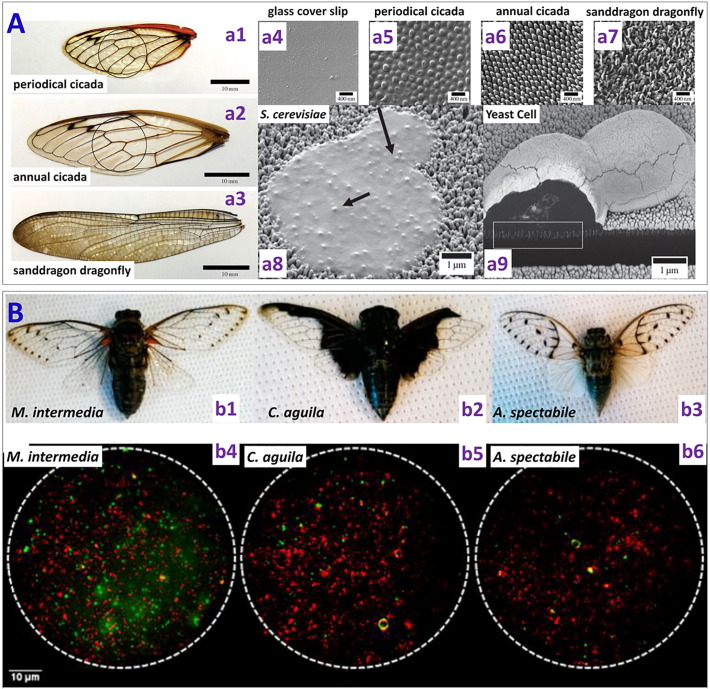
(A) (*Left*) Images of different types of wings used: (a1) Periodical cicada (*Magicicada* ssp.), (a2) Annual dog day cicada (*Tibicen* ssp.), and (a3) Common sanddragon dragonfly (*Pogomphus obscurus*). (*Top right*) (a4) Au-coated glass cover slip as control (a5) Periodical cicada wing showing hemispherical features with a mean diameter of 167 nm (a6) Annual cicada wing displaying spherically capped conical protrusions with a mean length of 183 nm and mean cap diameter of 57 nm (a7) Common sanddragon dragonfly wing displaying spherically capped cylindrical protrusions with a high aspect ratio which appear to be bundles of three to five smaller protrusions with a mean length of 241 nm and a mean bundle diameter of about 50 nm at the cap. (a8) High-resolution scanning electron micrographs representing morphology of ruptured yeast cell on the nanostructured common sanddragon dragonfly wing. (a9) SEM image of a ruptured yeast cell on the Annual dog day cicada wing. It shows the penetration of the nanostructures in to the yeast cell wall [reproduced with permission from Ref. [88]]. (B) Images of different types of cicada samples used (b1) *M. intermedia* (ME) (b2) *C. aguila* (CA) and (b3) *A. spectabile* (AY). Corresponding fluorescence microscopy images displaying the live cells (red) and the dead cells (green) on these surfaces [reproduced with permission from Ref. [89]].

From these models and related experimental studies, it has emerged that the bactericidal activity of a nanostructured surface depends on several parameters such as the size, shape and spacing/density of the nanostructures. Epstein et al. [87] discussed the effect of nanostructure geometry (spacing and aspect-ratio) on bacteria biofilm growth. Nowlin et al. [88] and Kelleher et al. [89] reported the eukaryotic and prokaryotic microorganism adhesion respectively on different types of cicada and dragonfly wings with different nanopillar height to width (*h/w*) ratios. The sanddragon dragonfly wing surface with the highest *h/w* ~ 4.6 (Fig. 9A) showed the highest bactericidal efficiency against *S. cerevisiae* (an eukaryotic microbe), causing more cell rupturing, as compared to the dog day (DD) annual cicada (Tibicen subspecies (ssp) *h/w* ~ 1.8) and the periodical cicada (*Magicicada* ssp, *h/w* ~ 0.5). It was thus suggested that allowing bacteria to adhere to the nanostructured surface and killing them physically could be a more effective strategy for antibacterial surface design than repelling the bacteria from the surface. Similarly, Kelleher et al. compared three cicada species with different nanopillar packing (or spacing): *M. intermedia* (height 241 nm, diameter 156 nm, pitch 165 nm), *C. aguila* (height 182 nm, diameter 159 nm, pitch 187 nm), and A. spectabile (height 182 nm, diameter 207 nm, pitch 251 nm) (Fig. 9B). Surfaces with tighter nanopillar packing and smaller feature (diameter) size (*M. intermedia* and *C. aguila*) showed higher bactericidal efficiency than the surface with a lower density and a bigger feature size (*A. spectabile*). These results suggest that the bactericidal efficiency can be tuned by the density and diameter of the nanostructures. However, they did not explain the underlying mechanism behind the higher killing efficiency of the surfaces with smaller diameter and higher density.

In addition to the physical effects of nanostructures, the chemical (e.g. hydrophobicity/hydrophilicity) and mechanical (e.g. pliability) effects may play important roles in conjunction with the physical ones on the bactericidal effects. The strength of adhesion between the bacteria and the nanostructured surface is a vital element in the nanostructures induced rupturing of the microbes. A larger adhesion force between the bacteria cell wall and the surface leads to a high probability of rupturing for a given nanostructure geometry [88]. Adhesion of the bacteria with the nanostructured surface depends on the hydrophobicity/hydrophilicity of the surface and the cell membrane composition. When challenged with the nanostructured surfaces, bacteria will try to settle on the nanostructured surfaces by increasing the contact area with multiple anchoring points. In this process of stretching, when the cell wall reaches a threshold limit of strain acting on it, the cell wall rupturing can take place. If the nanostructures present on the surface are pliable, they may bend and it is more difficult to attain the threshold strain for the stretched cell wall to become ruptured. This pliability might thus allow the bacteria to deform the nanostructures so that the microbes can settle and proliferate on “the bed of nails”.

## 9 Physical models for interactions of bacteria with the nanostructured surfaces

**Fig. 10:**
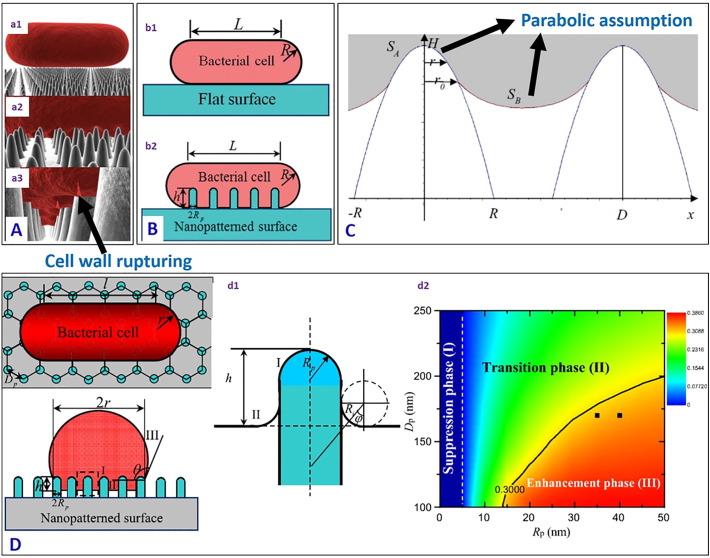
(A) Three-dimensional representation of the modelled interactions between a rod-shaped cell and the wing surface. As the cell (a1) comes into contact and (a2) adsorbs onto the nanopillars, the (a3) outer layer begins to rupture in the regions between the pillars [reproduced with permission from Ref. [92]] (B) Schematic illustration of the bacterial cell adhered to a (b1) flat surface and (b2) bacterial cell adhered to a cicada wing-like nanopatterned surface (L and R represent the length and radius of the bacteria respectively, h is the height of the nanopillar, *R*_p_ is the radius of the nanopillar) [reproduced with permission from Ref. [93]] (C) Side-elevation sketch map of a bacterial membrane adsorbing onto two neighbouring nanoridges, where *H* is the height of the nanoridge, 2*R* is the bottom width of the nanoridge, *S*_A_ denotes the contact area of the part of the bacterial membrane covering the nanoridge, *S*_B_ denotes the area of the suspended membrane, r_0_ is the distance from the dividing line to the x-axis, and *D* is the distance between two adjacent nanopillars [reproduced with permission from Ref. [94]] (D) (d1) Top view, cross-sectional view and enlarged view of bacteria membrane adhered to the surface with nanopillars in a hexagonal arrangement (*R*_p_ is the radius of the nanopillar, *D*_p_ is the distance between nanopillars, *L* and *R* represent the length and radius of the bacteria on the nanopatterned surface. *h* is the deformation depth, and *θ* the contact angle the bacteria cell membrane makes with the patterned surface). (d2) The phase diagram for the bacterial membrane stretching in the space of radius versus spacing of nanopillars (the colour bars indicate the values of the stretching degree of the bacterial membrane, with red corresponding to a high value and blue a low value) [reproduced with permission from Ref. [95]].

Pogodin et al. [92] developed a biophysical model to explain the interactions between the nanopillared cicada wing and bacteria, considering two sections of the bacteria cell wall: (a) the area in contact with the nanopillars, and (b) the area suspended between the nanopillars (Fig. 10A). The bacteria cell was assumed as a thin elastic layer as the dimension of the nanopillars (100 nm) on the cicada wing is an order of magnitude larger than the thickness of the cell wall (10 nm) and the curvature of the bacteria surface between the nanopillars was ignored. Due to the physical nanostructured topography present on the cicada wing, the bacteria membrane adsorbed on multiple nanopillars by enhancing the surface area of interaction. This led to a nonuniform stretching which in turn ruptured the membrane. Li et al. [93] studied the bactericidal mechanism of a nanostructured surface (of height *h* and radius *R*p in Fig. 10B) with a quantitative thermodynamic model by considering the free energy change of the bacteria cells (of length *L* and radius *R*), showing the difference in interaction of the bacteria cell wall with a flat surface and a nanostructured surface. The main contrast between the bacterial cell interaction with a flat surface and with a nanostructured surface is the contact area of adhesion and the deformation of the cell membrane in the adhesion area. The enhanced bactericidal efficiency of the nanostructured surface compared to a flat surface is attributed to the increase in the contact adhesion area (see Fig. 10B (b2)) which enhances the stretching strain of the membrane which leads to cell lysis when the stretching is sufficient, and could thus be promoted by increasing the surface roughness, and the radius and height of the nanostructures.

In a similar model for the cicada wing, Xue et al. [94] assumed a parabolic profile for the deformation of the bacterial membrane in the area both in contact with the nanopillars, and that hanging between the nanopillars (Fig. 10C). This was different from the study of Pogodin et al. [92] in which they ignored the curvature of the bacteria membrane hanging between the nanopillars. The combined role of gravity and van der Waals forces in rupturing the cell wall were considered, and it was shown that gram negative bacteria could be killed with a very high efficiency by the nanopillared wing surface. It was also suggested that bactericidal efficiency could be enhanced by sharp nanofeatures with large spacing, which is contrary to the findings of Kelleher et al. [89] who recommended tighter nanotextures packing for higher killing efficiency. Li et al. [95] considered the balance between the cell-nanostructured surface adhesion energy and the deformation energy of the cell membrane (Fig. 10D (d1)). They argued that the adhesion energy could be enhanced due to an increase in the contact area caused by the surface roughness, and at the same time the deformation energy could also be increased by nanopillars with a small radius. A phase diagram (the colour bars indicate the values of the stretching degree of the bacterial membrane, with red corresponding to a high value (an enhancement phase) and blue a low value (a suppression phase)) was devised to explain the interrelated effects of the nanopillar radius and spacing on the adhesion of bacteria on the nanostructured surface (Fig. 10D (d2)). We refer readers to these references for a more detailed mathematical description.

## 10 Cost-effective large-scale fabrication of nanostructured bactericidal surfaces

**Fig. 11:**
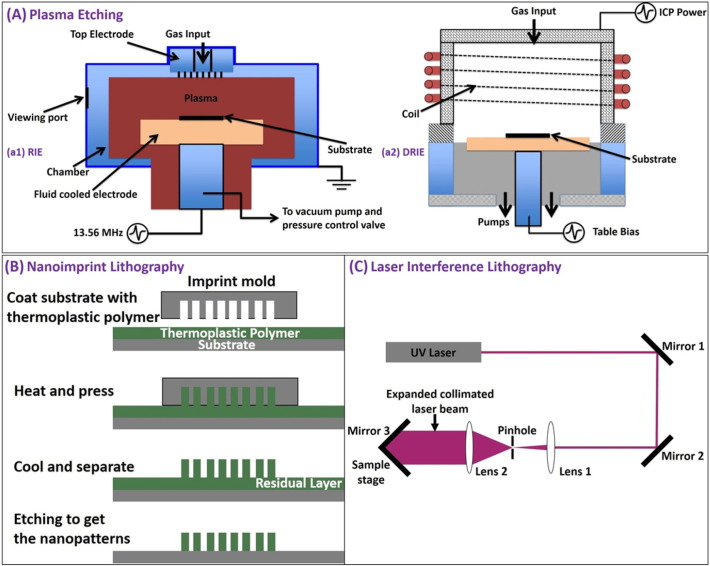
Nanostructure fabrication techniques: (A) Plasma etching. (a1) Reactive ion etching (RIE) is a plasma etching technique normally used in the semiconductor industry. The substrate is usually placed on a quartz or graphite plate. The gas required for etching is injected into the process chamber via the gas input present in the top electrode. Radio frequency (RF) plasma source is applied at the lower electrode which determines both the ion density and energy for etching. RIE is normally used to etch surface textures with depth < 1 μm. (a2) Deep reactive ion etching (DRIE) is a highly anisotropic etching process used to create deep penetration, through silicon via (TSVs) and trenches in wafers/substrates, typically with very high aspect ratios. To control the ion energy and ion density with more flexibility, separate RF (Table bias) and Inductively Coupled Plasma (ICP) generators are provided. (Source: Oxford Instruments) (B) Nanoimprint lithography (NIL) is a method for fabricating micro/nm scale patterns economically with high throughput and high resolution. NIL relies on direct mechanical deformation of the resist using an imprint mold unlike optical or electron beam lithographic approaches, which create pattern through the use of photons or electrons to modify the physical and chemical properties of the resist. It is therefore possible to achieve very good resolution beyond the limitations set by the diffraction of light or beam scattering that are observed in conventional lithographic techniques. Minimum feature size of the imprint mold determines the resolution of nanoimprint lithography. (C) Laser interference lithography (LIL) is a maskless technique. In this process a collimated laser beam is passed through a pinhole which only allows the central bright spot of the laser beam, and then expanded by Lens 3. Part of the expanded collimated beam falls directly on the photoresist-coated sample placed on the sample stage, which interferes with the other part reflected from Mirror 3 to create the interference pattern on the sample. The angle between the sample stage and the Mirror 3 can be adjusted to obtain desired interference patterns. The photoresist patterns produced with LIL provide the platform for further fabrication of different types of structures in the submicron scale.

Capability to fabricate biocompatible surfaces on a large scale via a cost-effective route is important practically and represents a technological challenge. Biocompatibility and selectivity of the textured material is essential for its use in medical devices for requirements of killing pathogenic bacteria while allowing the proliferation of mammalian cells. There are different techniques to fabricate nanostructured surfaces on variety of substrates [96], [97], [98], [99], [100], [101]. Fig. 11 shows the schematic of different fabrication methods commonly used, and Table 3 compares between different fabrication techniques in terms of their cost, complexity of the process and feasibility for large scale fabrication.

**Table 3:** Nanostructures fabrication techniques.

Method	Substrates applicable	Surface textures fabricated	Comments
Plasma etching	Silicon, glass, polymer, metals	Micro/nano pillars, nano wires, nano grass	Wafer scale large area fabrication possible, complex instrumentation, high cost
Nanoimprint lithography	Silicon, glass, polymer, metals	Micro/nano pillars, nano ridge	Feasibility of large scale fabrication depending on size of nanoimprint mold (fabrication area a few cm^2^), multi-step process, precision required for coating thermoplastic polymer, maintaining temperature and applied pressure, low cost
Laser interference lithography	Silicon, glass, polymer, metals	Nano ridges, nano pillars	Feasibility of large scale fabrication depending on power of laser and spot size of laser mold (fabrication area ~ cm^2^), complex setup, sensitive to white light exposure, vibration, high cost of laser source
Anodization	Metals	Nano spikes	Large area fabrication possible (fabrication area ~ cm^2^), simple process, low cost
Hydrothermal synthesis	Silicon, glass, polymer, metals	Nano rods, nano wires, nano needles	Large area fabrication possible (fabrication area in cm^2^), simple process, low cost

Serrano et al. [90] used oxygen plasma treatment on sutures to make nanotextured surfaces (see Table 2) by varying the etching time. These nanostructured sutures showed reduced bacterial adhesion and biofilm formation. This is a promising way to fabricate large area antibacterial surface as it can be realized at very low cost and can be applied to different polymer surfaces and geometry. Diu et al. [64] used a hydrothermal method for fabrication of Cicada wing inspired nanostructured (Brush and niche type, see Table 2) titania surfaces for potential dental and orthopaedic implant applications, and the obtained brush and niche type nanostructures both showed excellent bactericidal efficacy against pathogenic bacteria, while allowing the growth of mammalian cells.

Wu et al. [91] reported a template electrodeposition technique to fabricate gold nanopillars, nanorings and nanonuggets (see Table 2) on tungsten reference substrate. In this method, tungsten and aluminium thin films were first deposited on silicon substrates. Then the nanoporous alumina template was generated by anodization of the top layer aluminium. Reference tungsten substrate with nanoscale roughness was obtained by dissolving the alumina nanoporous template. Au nanopillars were then electrodeposited within the nanoporous template and the aluminium template was removed. Similarly, Au nanorings and clusters of nanopillars were obtained by modifying the structure of the tungsten layer. All of these surfaces were tested against gram positive *S. aureus* bacteria, showing excellent bactericidal performance, as qualitatively validated by scanning electron microscopy and fluorescence microscopy images, with cell proliferation experiments also carried out to evaluate the antibacterial performance quantitatively. The template electrodeposition technique may not be cost effective for fabrication on a small scale. However, it can be economical for manufacturing on a large scale.

Ozkan et al. [102] fabricated nanostructured superhydrophobic antibacterial surface by combining PDMS and Cu nanoparticles via AACVD (aerosol-assisted chemical vapour deposition) (sample dimension - 14 cm × 4.5 cm × 0.5 cm). Static water contact angles as high as 151° were obtained on the fabricated surface, the surface showed excellent bactericidal property, killing gram positive *S. aureus* in 1 h and gram negative *E. coli* in only 15 min. These techniques described above can be used as the basis for fabricating large area cost-effective bactericidal surfaces and can be used for practical applications.

## 11 Summary

Antimicrobial resistance has become an urgent global challenge and smart alternative solutions are needed to tackle bacterial infections. Bacteria differ in shape, size, cell wall thickness, outer membrane composition and indeed other characteristics. A number of insects and plants with sharp nanostructures on their surfaces can kill bacteria by physically rupturing/stretching the bacteria cell wall via the contact killing mechanism. This review aims to highlight our current understanding on how natural and bioinspired nanostructured surfaces interact with bacteria cell wall membranes, and these kinds of nanostructured bactericidal surfaces have the potential to be incorporated in many biomedical and industrial applications as an alternative to, or a synergistic combination with, chemical bactericidal mechanisms. Bactericidal efficiency of nanostructured silicon, nanostructured titania, nanostructured polymer surfaces has already been tested against different pathogenic bacteria, and these nanostructures represent a wide range of shape, size, density, rigidity. How these physical parameters can be optimized to enhance the bactericidal efficiency remains a challenge. Different fabrication techniques have been briefly discussed, with the focus on their feasibility for cost effective, large area production of nanostructured surfaces, which is an important consideration when we consider employing the contact killing mechanisms as part of our material design. Further considerations involve selectivity of the nanostructured surfaces, i.e. benign or even functionally active towards mammalian cells but hostile towards bacteria. Though physical killing of bacteria has been demonstrated on different nanostructure morphologies, there is no clear generic guideline which holds true for all the bacteria and for all the substrates with different mechanical and chemical properties. Different models give preference to different pitches: (1) Lower pitches preferred by bending energy models; and (2) higher pitches preferred by stretching based models. Too high a pitch may in fact lead to growth of bacteria in between the pillars. It also appears that a certain minimum aspect ratio is required for the nanotexture, as otherwise the cell would be able to “sense” its topology. Finding an optimized nanotopography in terms the size, shape, aspect ratio, and density, which should be tuned for different sizes and types of bacteria, remains a significant scientific challenge. It is envisaged that developments in the design, fabrication, optimisation, and mechanistic understanding of bactericidal efficacy of such nanostructured bactericidal surfaces present many opportunities for further investigations and may serve as an effective strategy in combating pathogenic bacteria and rising to the challenge of antimicrobial resistance facing us.
